# Knowledge of Epilepsy and Seizure First Aid Among Teachers in Eastern Province, Saudi Arabia

**DOI:** 10.7759/cureus.33418

**Published:** 2023-01-05

**Authors:** Nora AlMuslim, Mohammed Aldawood, Ibrahim Almulhim, Rabab Alhaddad, Ashiq AlQahtani, Abdullah Almubarak

**Affiliations:** 1 College of Medicine, Imam Abdulrahman University, Dammam, SAU; 2 College of Medicine, King Faisal University, Al-Ahsa, SAU

**Keywords:** eastern province, saudi arabia, teachers, epilepsy, awareness

## Abstract

Background: Epilepsy is one of the most common neurological disorders among patients, with a high prevalence in adults and children in Saudi Arabia. It can have a negative impact on a child's health, behavior, and academic performance, as well as their mental health. As a seizure attack can happen in school and the teachers will be the first health providers, preventing complications and ensuring student performance, development of social skills, and future employment can be significantly impacted by a teacher's awareness and attitudes towards epilepsy.

Methods: A descriptive cross-sectional study was conducted in Eastern Province, Saudi Arabia, targeting teachers in schools of all educational levels using a self-administrated validated questionnaire in Arabic. A total of 423 teachers fulfilling the inclusion criteria were included. Teachers' ages ranged from 18 to more than 50 years with mean age of 42.6 ± 9.3 years old. Exactly 261 (61.7%) teachers were females and 400 (94.6%) were Saudi.

Results: We defined acceptable level of awareness as the ability to recognize seizure phenomena (if they have witnessed one before) as a medical neurological problem (as opposed to attributing it to religious and/or superstitious beliefs, etc.) with ability to respond promptly with proper first aid required when encountering a seizure (turn on one side, avoid placing items in mouth, etc). Exactly 163 (38.5%) had good overall knowledge level while 260 (61.5%) had poor knowledge regarding epilepsy.

Conclusion: Our research found that teachers have poor knowledge about epilepsy, wherein 38.5% had good overall knowledge level while 61.5% had poor knowledge regarding epilepsy. However, only a 9.2% had first-aid training, which suggests that knowledge and practice of epilepsy first aid needs to be improved via public awareness campaigns and first-aid training courses in schools.

## Introduction

Epilepsy is known to be one of most common disorders among children [1]. It is defined as recurrent seizures that happen as a result of a paroxysmal excessive electrical discharge of cortical neurons [2]. The prevalence of epilepsy has been estimated to be greater than 50 million [3]. The epilepsy prevalence in Saudi Arabia is estimated as 6.54 per 1,000 among children and adults [4]. Furthermore, seizure attack can happen in school, and the teachers will be the first health providers [5]. Epilepsy can have a negative impact on a child's health, behavior, and academic performance, as well as their mental health. The child's quality of life and potential adult roles will be greatly impacted by the school years. Preventing complications and ensuring student performance, development of social skills, and future employment can be significantly impacted by a teacher's awareness and attitudes towards epilepsy. Despite the fact that teachers have a significant impact on the lives of these children, very few researches have been conducted in Saudi Arabia to assess these issues [6]. Similar studies in Japan, Kuwait and Khartoum have showed that teachers did not have enough knowledge about epilepsy and seizures first aid [7-9]. On the other hand, a study in Jordan has shown that teachers have moderate knowledge [10]. Studies in Saudi Arabia, specifically in Riyadh and Khamis Mushait, have revealed that teachers have adequate knowledge [11,12]. Conversely, teachers in Tabuk, Arar, and Makkah have insufficient knowledge about the studied issue [13-15]. To the best of our knowledge, no similar studies have been conducted in Eastern Province, Saudi Arabia. Our study's aim is to assess the awareness of epilepsy and its first aid among teachers at all educational levels in Eastern Province, Saudi Arabia. 

## Materials and methods

The study was approved by the Research Ethics Committee at King Faisal University in September 2022 (KFU-REC-2022-SEP-ETHICS127) . A descriptive cross-sectional study was conducted in Eastern Province, Saudi Arabia, from September to October 2022. Our target population was female and male teachers in both private and public schools of Eastern Province, Saudi Arabia, regardless of the educational level.

The sample size was 402 and was calculated using the Richard Geiger equation. A self-administrated validated questionnaire in Arabic was used. The questionnaire was taken from a study that assessed knowledge about seizure first aid among teachers in Jeddah, Saudi Arabia. The questionnaire had two sections, in which the first assessed demographics (city, age, gender, nationality, and qualification), while the second covered the knowledge of epilepsy, seizure, and its first aid among teachers.

It consisted of six questions that evaluated the participants’ knowledge, where the responses were added together to construct a knowledge score. Based on the number of correct answers, teachers' knowledge categories were classified into good (five to six correct answers), moderate (three to four correct answers), and poor (one to two correct answers).

The questionnaire was distributed electronically to the teachers who were encouraged to share the form with their colleagues using a snowball sampling technique. Data entry was done using Microsoft Excel 2016. The statistical analysis was performed using a Statistical Package for the Social Sciences (SPSS) software, version 25 (IBM Corp, Armonk, USA) through the application of a chi square analysis. Statistical significance was determined, using P values with a threshold less than .05 and a confidence interval of 95%.

Confidentiality of the participants was insured, and all the collected information in this study was used for scientific purposes only.

After collecting the data, it was reviewed and then fed to SPSS version 21 . All statistical methods used were two tailed with alpha level of 0.05 considering significance if P value was less than or equal to 0.05. Regarding knowledge, each correct answer was given a one-point score. Overall knowledge level regarding epilepsy was assessed through summing up discrete scores for different correct knowledge items. The overall knowledge score was categorized as poor if a participant's score was less than 60% of the overall score and as good if the participant's score was 60% or more of the overall score. Descriptive analysis was carried out through prescribing frequency distribution and percentage for study variables including teacher's personal data, experience years, training, and experiencing seizures. Also, knowledge items were tabulated while overall knowledge level was graphed. Cross tabulation for showing distribution of participants’ overall knowledge level by their personal data and experience years was done with Pearson chi-square test for significance and exact probability test if there were small frequency distributions.

## Results

A total of 423 teachers who fulfill the inclusion criteria were included. Teachers' ages ranged from 18 to more than 50 years with mean age of 42.6 ± 9.3 years old. Exactly 261 (61.7%) teachers were female and 400 (94.6%) were Saudi. As for qualification, 399 (94.3%) had a bachelor's degree and 24 (5.7%) had a postgraduate degree. Teaching experience for more than 10 years was reported among 303 (71.6%) teachers and 72 (17%) had experience of one to five years. A total of 39 (9.2%) were trained on how to deal with epileptic seizures and 117 (27.7%) had previously witnessed one of their students' seizures (Table 1).

**Table 1:** Bio-demographic data of teachers who participated in the study in Eastern Province, Saudi Arabia

Bio-demographic data	No.	%
Age in years		
< 30	49	11.6%
30-39	80	18.9%
40-49	187	44.2%
50+	107	25.3%
Gender		
Male	162	38.3%
Female	261	61.7%
Nationality		
Saudi	400	94.6%
Non-Saudi	23	5.4%
Qualification		
Bachelor's	399	94.3%
Master's	22	5.2%
PhD	2	.5%
Teaching stage		
Primary	142	33.6%
Intermediate	110	26.0%
Secondary	171	40.4%
Teaching experience years		
1-5	72	17.0%
6-10	48	11.3%
> 10	303	71.6%
Did you get any training on how to deal with Epileptic seizures?		
Yes	39	9.2%
No	384	90.8%
Have you witnessed a seizure on one of your students before?		
Yes	117	27.7%
No	306	72.3%

Teachers' knowledge and awareness regarding epilepsy in Eastern Province, Saudi Arabia

Exactly 87.5% of the study participants knew that epilepsy is a neurological disorder and 65.5% know that there is a treatment for epilepsy. A total of 61.2% reported that drugs for epilepsy treatment did not cause addiction. Also, 52% reported that they will ensure the patients safety and ask for help if one of their students had a seizure attack, while 64.8% reported that they will lay the student on his side and ask for help after a seizure ends. When teachers were asked about when to transport the student to a hospital, 17% stated if a seizure lasted for more than five minutes, 11.6% reported if the seizure reoccurred and the student did not gain consciousness. However, 27% will transport the student to hospital when seizure last for more than five minutes or reoccur and the student did not gain consciousness (Table 2).

**Table 2:** Teachers' knowledge and awareness regarding epilepsy in Eastern Province, Saudi Arabia

Knowledge items	No.	%
Causes of epilepsy		
Neurological disorder	370	87.5%
Psychological disorder	46	10.9%
Demonic possession	7	1.7%
Is there a treatment for epilepsy?		
Yes	277	65.5%
No	146	34.5%
Do epilepsy treatment drugs cause addiction?		
Yes	164	38.8%
No	259	61.2%
What is your response if one of your students had a seizure attack?		
Ensure the patients safety and ask for help	220	52.0%
Read the Quran	8	1.9%
Open his mouth and put gauze in it	195	46.1%
What do you do after a seizure ends?		
Lay the student on his side and ask for help	274	64.8%
Try to wake him up	60	14.2%
Read the Quran	6	1.4%
Wash his face with water and give him water to drink	83	19.6%
When do you have to transport the student to a hospital?		
Immediately if a seizure occurred	53	12.5%
If a seizure continued for more than 5 minutes	72	17.0%
If a seizure continued for more than 10 minutes	31	7.3%
If a seizure continued for more than 20 minutes	17	4.0%
If the seizure reoccurred and the student didn’t wake up	49	11.6%
Both A & B	84	19.9%
Both B & E	117	27.7%

Regarding factors associated with teachers' overall knowledge level regarding epilepsy in Eastern Province, Saudi Arabia, exactly 50% teachers with a PhD had good knowledge level in comparison to 38.8% of others with a bachelor's degree with recorded statistical significance (P=.048). All other factors showed insignificant relation with teachers knowledge level (Table 3).

**Table 3:** Factors associated with teachers' overall knowledge level regarding epilepsy in Eastern Province, Saudi Arabia P: Pearson X2 test; * P < 0.05 (significant); †: Exact probability test

Factors	Overall knowledge level				P value
	Poor		Good		
	No.	%	No.	%	
Age in years					.528
< 30	30	61.2%	19	38.8%	
30-39	46	57.5%	34	42.5%	
40-49	122	65.2%	65	34.8%	
50+	62	57.9%	45	42.1%	
Gender					.347
Male	95	58.6%	67	41.4%	
Female	165	63.2%	96	36.8%	
Nationality					.346
Saudi	248	62.0%	152	38.0%	
Non-Saudi	12	52.2%	11	47.8%	
Qualification					.049*†
Bachelor	244	61.2%	155	38.8%	
Master	15	68.2%	7	31.8%	
PhD	1	50.0%	1	50.0%	
Teaching stage					.701
Primary	90	63.4%	52	36.6%	
Intermediate	69	62.7%	41	37.3%	
Secondary	101	59.1%	70	40.9%	
Teaching experience years					.539
1-5	43	59.7%	29	40.3%	
6-10	33	68.8%	15	31.3%	
> 10	184	60.7%	119	39.3%	
Did you get any training on how to deal with Epileptic seizures?					.496
Yes	22	56.4%	17	43.6%	
No	238	62.0%	146	38.0%	
Have you witnessed a seizure on one of your students before?					.985
Yes	72	61.5%	45	38.5%	
No	188	61.4%	118	38.6%	

Overall knowledge level regarding epilepsy among teachers who participated in the study in Eastern Province, Saudi Arabia

**Figure 1:**
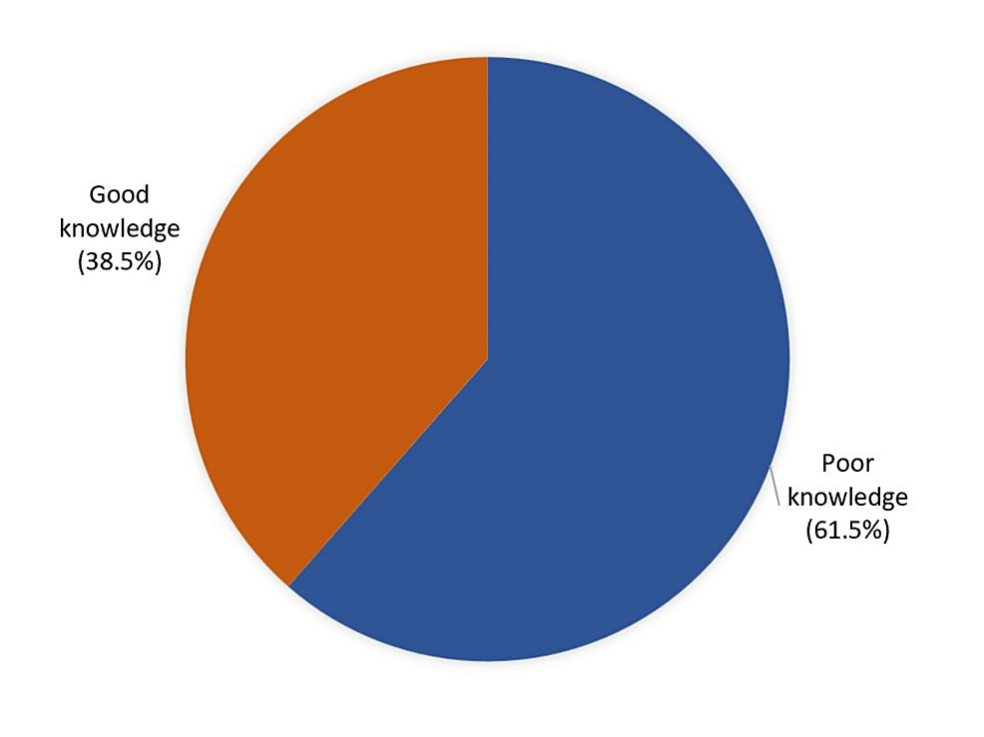
Overall knowledge level regarding epilepsy among teachers who participated in the study in Eastern Province, Saudi Arabia

Exactly 163 (38.5%) had good overall knowledge level while 260 (61.5%) had poor knowledge regarding epilepsy (Figure 1).

## Discussion

This study is aimed to assess the knowledge of epilepsy and seizure first aid among teachers at all educational levels in Eastern Province, Saudi Arabia. Patients with epilepsy may encounter factors that affect their quality of life in their daily lives, including the need for emergency first aid [16]. Teachers should therefore be able to handle this disease and reduce its impact on their students.

Our research revealed that more than half had an inadequate understanding of epilepsy. This is consistent with some studies conducted throughout Saudi Arabia that revealed that majority of people do not have enough understanding of epilepsy [13-15]. However, some studies in Jordan and Jeddah showed schoolteachers having moderately good knowledge and attitude toward epilepsy [10,17]. In addition, the majority of our participants were aware that epilepsy is a neurological condition; the percentage was nearly equal to that in previous studies done in Jordan and Makkah and higher than that reported in Kuwait and Sudan [8-10,15]. Our study results support our theory of lack of knowledge and response regarding epilepsy and seizure first aid, which indicates the importance of initiating proper educational programs about common diseases in the area.

Additionally, only 1.7% of our participants believed that demonic possession is the cause of epilepsy, which is a significant improvement over a prior study conducted in Saudi Arabia, which found that nearly half of the study population believed that possession may be the cause of epilepsy [18]. This finding demonstrates an improvement in teachers' understanding of epilepsy, which may be a result of increased awareness campaigns in addition to the Ministry of Health's efforts to make scientific resources accessible to the general population. More educational initiatives, however, can dispel these misconceptions and advance public awareness of epilepsy.

Although the vast majority of participants did not receive first-aid training, nearly half of them correctly answered questions on how they would react during a seizure attack. Numerous studies conclusively show that there are not enough educators with first aid training [8-10,15,17]. This knowledge could be the result of searching on the internet or from past experiences that made them eager to search and know more about epilepsy, for that reason they should get the information from the right source based on guidelines. Regardless of their knowledge to respond during a seizure attack, only a third of our participants knew all conditions that necessitate hospital transfer. This was also reported by a previous study done in Jeddah [17]. This result indicates more knowledge for teachers is needed to know when transferring students to the hospital is crucial.

Our study showed a significant relationship between knowledge level and teacher's educational degree, where half of the PhD teachers had good knowledge in comparison to only 38% of bachelor's-degree teachers. This result is similar to the studies conducted in Arar and Jeddah [13,17].

Our study demonstrated similar results to the studies conducted in Arar and Jeddah [13,17]. Our study did not show a significant association between knowledge level, gender, and age. This shows how Saudi Arabia's working environment and educational background is similar, even if the study is conducted in different regions.

While this article relies on the strength of good statistical analysis and literature review, which resulted in a well-rounded discussion and result presentation, there may have been some unavoidable limitations to the study. The main limitation is that our study result does not reflect other larger cities and rural areas who are expected to be less knowledgeable and more towards superstitious beliefs about epilepsy.

## Conclusions

This study intended to assess awareness of epilepsy and its first aid among teachers at all educational levels in Eastern Province, Saudi Arabia. Our study showed that teachers have poor knowledge about epilepsy, which shows that 38.5% had good overall knowledge level while 61.5% had poor knowledge regarding epilepsy. However, only a 9.2% had first-aid training, which suggests the need for raised awareness to increase knowledge and practice needed for first responder training in schools. Further study on the knowledge of epilepsy in Saudi Arabia is recommended.
